# The Beneficial Effects of Allicin in Chronic Kidney Disease Are Comparable to Losartan

**DOI:** 10.3390/ijms18091980

**Published:** 2017-09-16

**Authors:** Ehécatl Miguel Ángel García Trejo, Abraham Said Arellano Buendía, Omegar Sánchez Reyes, Fernando Enrique García Arroyo, Raúl Arguello García, María Lilia Loredo Mendoza, Edilia Tapia, Laura Gabriela Sánchez Lozada, Horacio Osorio Alonso

**Affiliations:** 1 Renal Physiopathology Laboratory, Depterment of Nephrology, Instituto Nacional de Cardiología “Ignacio Chávez”, Mexico City 14080, Mexico; dr.ehecatl@hotmail.com (E.M.Á.G.T.); neoabraham_7@hotmail.com (A.S.A.B.); tuzome_sanrey23@hotmail.com (O.S.R.); jonibertojr@hotmail.com (F.E.G.A.); ediliatapia@hotmail.com (E.T.); lgsanchezlozada@gmail.com (L.G.S.L.); 2 Departamento de Genética y Biología Molecular, Centro de Investigación y de Estudios Avanzados-IPN, Mexico City 07360, Mexico; raularguellogarcia@yahoo.com; 3 Histopathology Laboratory, Research Subdivision, School of Medicine, Universidad Panamericana, Donatello 43, Mexico City 03910, Mexico; lloredo@up.edu.mx

**Keywords:** allicin, hypertension, chronic kidney disease, oxidative stress, losartan

## Abstract

Recent studies suggest that allicin may play a role in chronic kidney disease (CKD), reducing hypertension and oxidative stress and improving renal dysfunction. In the present study, CKD was induced by 5/6 nephrectomy and the animals were divided into four treatment groups as follows: control (C), CKD, CKD+allicin (40 mg/kg pathway oral) (CKDA), and CKD+Losartan (20 mg/kg) (CKDL). After CKD induction, the rats developed hypertension from week 3 to the end of the study. This was associated with increased creatinine and blood urea nitrogen (BUN) levels in serum, increased albuminuria, increased urinary excretion of *N*-acetyl-β-d-glucosaminidase (NAG), increased nephrin expression, and incrased histological alterations in the cortex. The levels of angiotensin receptors and endothelial nitric oxide synthase (eNOS) were decreased in the renal cortex from the CKD group. Otherwise, lipid and protein oxidation were higher in the CKD group than in the control group. A disturbance was observed in the expression levels of the nuclear factor erythroid 2-related factor 2/Kelch ECH associating protein 1 system (Nrf2/keap1) and the antioxidant enzymes catalase, superoxide dismutase, and heme oxygenase-1. Allicin or losartan treatments relieved renal dysfunction, hypertension, and oxidative stress. In addition, both treatments showed the same efficacy on the expression of angiotensin receptors, the nephrin, Nrf2/keap1 pathway, and eNOS. Further in silico analyses suggest that allicin and losartan could have a common mechanism involving interaction with AT1 receptors. Allicin showed antihypertensive, antioxidant, and nephroprotective effects. The beneficial effects showed by allicin are similar, or even better, than those of losartan. In fact, the effect of allicin on blood pressure and renal function is comparable to reductions seen with losartan, a prescription drug commonly used as a first-line therapy.

## 1. Introduction

Hypertension is the leading cause of chronic kidney disease (CKD) and end-stage renal disease (ESRD), contributing to the disease itself or, most commonly, contributing to its progression. Likewise, hypertension is reported as a consequence of kidney injury [1]. In the United States, approximately 30% of incident ESRD cases are attributed to hypertension [2]. Hypertension is highly prevalent in patients with CKD, playing a role in the high cardiovascular morbidity and mortality of this particular population. In fact, the risk of cardiovascular death in this patient group is greater than the risk of progression to ESRD. Conversely, CKD is the most common form of secondary hypertension, and mounting evidence suggests that it is an independent risk factor for cardiovascular morbidity and mortality [3,4,5].

In CKD patients, the goal of hypertension management involves not only cardiovascular protection by lowering blood pressure (BP) to the appropriate level but also slowing the progression of kidney disease. The latter often includes the management of proteinuria, which is itself associated with both the risk of cardiovascular disease and progression to ESRD [6,7]. Therefore, it is of great importance to choose an appropriate antihypertensive agent for patients with CKD.

Current therapies include antihypertensive drugs such as angiotensin II-converting enzyme inhibitors (ACEI) or angiotensin II receptor blockers (ARBs), both as first choice drugs. These antihypertensives effectively reduce hypertension and cardiovascular diseases, providing nephroprotection and thereby slowing the progression of CKD [7,8]. In addition, these particular drugs have the ability to reduce cardiovascular morbidity and mortality.

In recent years, studies on the beneficial effects of extracts of *Allium sativum* L. (garlic) in the treatment of chronic diseases such as diabetes and hypertension have been carried out [9,10,11,12]. The use of garlic as an antihypertensive it is not well established, probably because the active substances responsible for the therapeutic effects are not known with certainty. Among the active constituents in garlic, one major component is allicin (thio-2-propene-1-sulfinic acid *S*-allyl ester), which is formed from the stable precursor *S*-allyl cysteine-*S*-oxide (alliin) by the action of the enzyme alliinase when garlic cloves are crushed or macerated [13]. Allicin has been shown to lower blood pressure in hypertensive patients, as well as in hypertensive rats [14,15,16,17].

CKD places the patient into the highest cardiovascular risk level, irrespective of traditional cardiovascular (CV) risk factor stratification. In this regard, we recently reported that allicin treatment in rats with subtotal nephrectomy improves cardiac function and ameliorates oxidative stress and hypertension [18], supporting the hypothesis that allicin could be important as a complementary and/or alternative therapy to reduce the progression of CKD and decrease the risk of cardiovascular disease.

To position our findings in line with translational research, our aim in the present study was to compare the effects of allicin with losartan, a first-line and well-known antihypertensive drug, in experimental CKD therapy with particular emphasis on kidney function and hypertension.

## 2. Results

### 2.1. Physiological Data

All experimental groups had a similar mean body weight at the beginning of the experiment. Nevertheless, six weeks later, the body weight of the CKD group was lower compared to that of the control group (Table 1). Interestingly treatment with allicin or losartan stimulated weight gain at higher levels than the untreated group (Table 1), and weight differences were not found between losartan and allicin treatment (Table 1).

Renal function was evaluated by the determination of creatinine and blood urea nitrogen (BUN) levels in serum and creatinine clearance. As expected in this experimental model of CKD, the subtotal nephrectomy resulted in a renal dysfunction status at six weeks of follow-up, which was evidenced by the significant increase in creatinine and BUN levels in serum, as well as the reduction in creatinine clearance (Table 1). The treatment with allicin or losartan showed beneficial effects. Thus, the body weight and creatinine clearance were increased with both treatments with respect to the untreated CKD rats. On the other hand, the creatinine and BUN levels in serum were decreased with allicin or losartan when compared with the untreated group (Table 1). Treatments with allicin or losartan showed the same efficacy on these parameters; however, losartan was slightly more efficient than allicin in reducing creatinine levels in serum (Table 1).

### 2.2. Systolic Blood Pressure (SBP)

**Figure 1:**
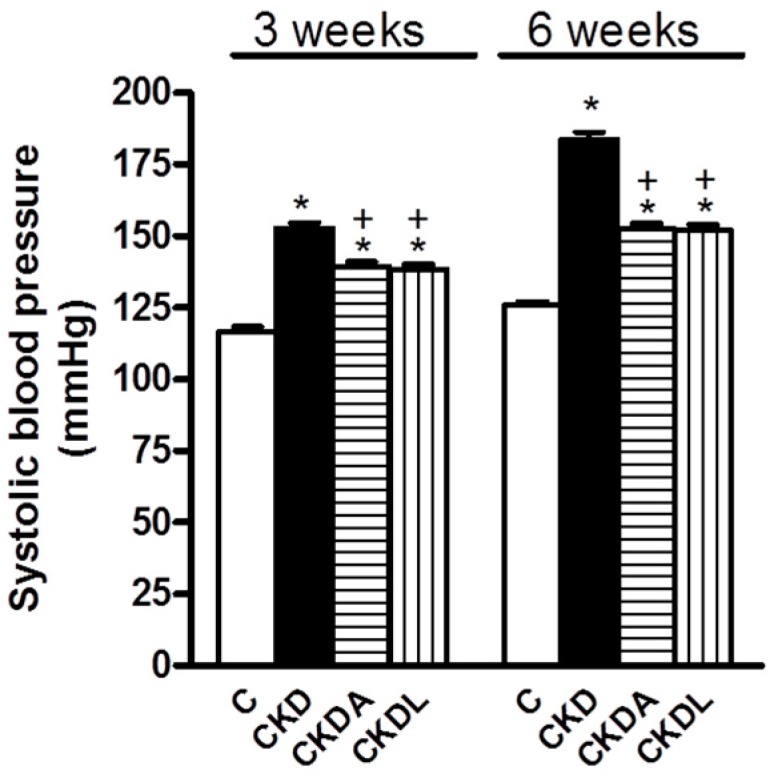
Systolic blood pressure at three and six weeks of follow-up. Control, C; chronic kidney disease, CKD; chronic kidney disease, allicin-treated, CKDA; and chronic kidney disease, losartan-treated, CKDL. Values represent mean ± SEM, *n* = 6. * *p* < 0.05 versus C; ^+^ *p* < 0.05 versus CKD.

The progression of CKD is associated with high blood pressure, which may develop even at early stages in the course of the disease, and has been associated with worsening renal function as well. Rats with CKD induction developed high blood pressure, which was evident at three weeks of follow-up and was severe at six weeks when compared with the control group (Figure 1). Three weeks after CKD induction, there was an increase in the SBP in the CKD group by approximately 30% with respect to the control group (Figure 1).

In the CKD allicin-treated group, there was a significant decrease (≈10 mmHg) in the SBP as compared to the untreated CKD group (Figure 1). Compared with losartan, the allicin treatment did not show significant differences (Figure 1).

At six weeks of follow-up, rats with CKD developed severe systemic hypertension compared with the control group (183.8 ± 2.62 versus 125.8 ± 1.36 mmHg, respectively) (Figure 1). The allicin treatment prevented the increase in SBP, causing it to end at 146 mmHg, almost 40 mmHg lower than that of the untreated group (Figure 1). Despite the antihypertensive effects of allicin and losartan, they did not achieve blood pressure levels similar to those recorded in the control group. Compared with losartan, the effect of allicin treatment did not show statistically significant differences (Figure 1). Therefore, losartan and allicin showed equal effectiveness in hypertension reduction.

### 2.3. Markers of CKD Progression

Blood pressure control is associated with renal protection, thereby slowing CKD progression. Amongst the most significant markers of CKD progression are albuminuria and tubular parameters such as urinary excretion of *N*-acetyl-β-d-glucosaminidase (NAG). Thus, to address the issue of blood pressure control and the progression of CKD we assessed albuminuria and nephrin as markers of glomerular injury and NAG excretion as an indicator of tubular damage.

**Figure 2:**
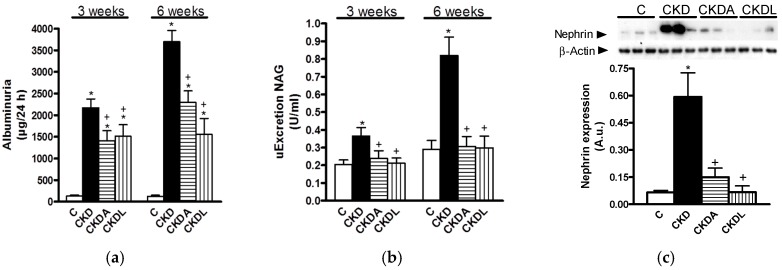
Analysis of markers of renal injury: (**a**) Albuminuria; (**b**) urinary excretion of *N*-acetyl β-d-glucosaminidase (NAG); and (**c**) semi-quantitative analysis of nephrin protein expression in the kidney. Control (C); chronic kidney disease (CKD); CKD, allicin-treated (CKDA); and CKD, losartan-treated (CKDL). Data are expressed as mean ± SEM, *n* = 6. * *p* < 0.05 versus C; ^+^ *p* < 0.05 versus CKD.

At three weeks of follow-up, the CKD group showed higher albuminuria compared to the control group (Figure 2a). Albuminuria was aggravated at the end of the study. Additionally, the urinary excretion of NAG showed a similar trend, which suggested a time-dependent CKD progression at the glomerular and tubular levels (Figure 2b).

The allicin or losartan treatments showed renal protection at the glomerular and tubular levels (Figure 2). Although the losartan and allicin treatments were equally effective, neither was able to completely prevent albuminuria reaching those values observed in the control group (Figure 2).

Since our results showed that hypertension and albuminuria were severe at the end of study, we evaluated the integrity of the glomerular filtration barrier (i.e., nephrin expression) at this time. Nephrin gene expression was assessed by Western blot assays of the kidney cortex. As can be seen in Figure 2c, nephrin expression was increased in the CKD group when compared with the control group and the allicin or losartan treatments attenuated this effect.

### 2.4. Histopathological Study

**Figure 3:**
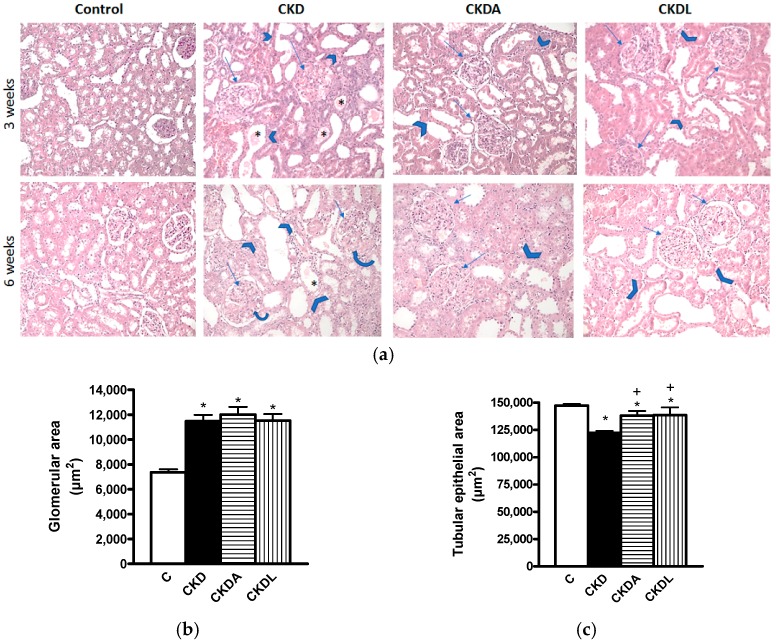
Representative microphotographs of the kidney cortexes of animals subjected to subtotal renal ablation for the induction of CKD at three weeks (upper images) and six weeks (lower images) of follow-up (**a**). Quantitative analysis of glomerular area (**b**); and tubular epithelial area (**c**) at six weeks of follow-up. Controls (C) show no histological abnormalities. The chronic kidney disease group (CKD) shows glomerular (arrows) changes as mesangial expansion and fibrosis and shows tubular (arrow heads) lesions as lumen size augmentation, epithelial thinning, and the presence of hyaline casts (*). The allicin-treated (CKDA) and losartan-treated (CKDL) groups’ images demonstrate a clear amelioration of these glomerular and tubular changes. (HE, original magnifications 100×). Data are expressed as mean ± SEM, *n* = 6. * *p* < 0.05 versus C; ^+^ *p* < 0.05 versus CKD.

Hematoxylin and eosin (HE) staining revealed very important histological changes in the renal cortex of animals from the CKD group in comparison to the unaltered architecture in the kidneys of the control group. The glomerular changes at three weeks of follow-up were characterized by a size increase, mesangial expansion and fibrosis, Bowman’s capsule adhesions, capillary occlusion, and decreased cellularity (Figure 3a). At six weeks post-renal ablation, these alterations were augmented, except for the glomerular size, which remained approximately the same. The tubular alterations at three weeks of CKD induction consisted of lumen expansion, epithelium thinning, and hyaline casts. The first two lesions were increased at six weeks of follow-up (Figure 3a). As shown in Figure 3a, the glomerular and tubular histological changes at three and six weeks of follow-up were diminished in both treated groups (CKDA and CKDL).

Additionally, at six weeks, we quantified the glomerular and tubular areas. Our results demonstrated an increase in glomerular area in the CKD group compared with the control group (Figure 3b). In contrast, the epithelial tubular area of the CKD group was decreased in comparison to that of the control group (Figure 3c). The decrease of this parameter is indicative of atrophy of the tubular epithelium. The allicin and the losartan treatments in CKD groups greatly attenuated these histologic alterations (Figure 3b,c).

**Figure 4:**
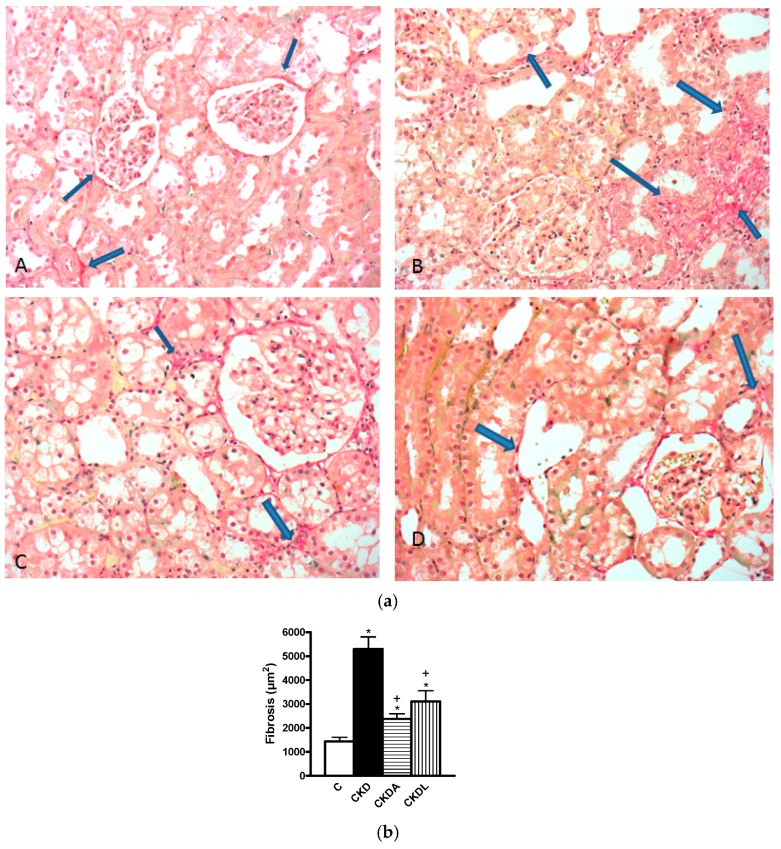
Evaluation of fibrosis. (**a**) Red Sirius stained representative photomicrographs. (**A**) Control shows collagen in red only in the Bowman capsule and in the vicinity of an artery; (**B**) Extense red areas (fibrosis) in the cortical interstitium; Alicin (**C**) and losartan (**D**) treated animals respectively, depict a very important decrease in the red stained areas in comparison with B. Arrows localized collagen in the different images. (Original magnifications 200×). (**b**) Quantitative analysis. Control (C); chronic kidney disease (CKD); CKD, allicin-treated (CKDA); and CKD, losartan-treated (CKDL). Data are expressed as mean ± SEM, *n* = 6. * *p* < 0.05 versus C; + *p* < 0.05 versus CKD.

At six weeks of follow-up, we assessed the fibrosis by Sirius Red staining. The fibrosis assessment was significantly increased in the cortex of the CKD group in comparison to that of the control group (* *p* < 0.05). There was less fibrosis in the group with CKDA and CKDL when compared with the untreated group (^+^ *p* < 0.05) (Figure 4).

### 2.5. Expression of Angiotensin II Receptors in Kidney Cortex

**Figure 5:**
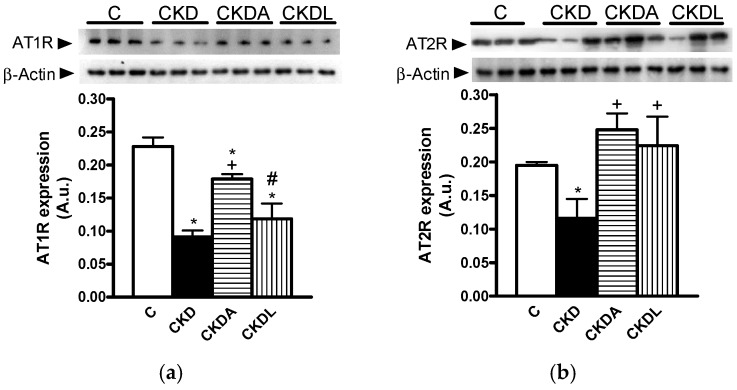
Chronic kidney disease dysregulated angiotensin II receptors in the renal cortex. (**a**) Angiotensin II type 1 receptor (AT1R) and (**b**) Angiotensin II type 2 receptor (AT2R). Representative immunoblot and quantitative analysis of relative protein expression. Control, C; chronic kidney disease, CKD; chronic kidney disease, allicin-treated, CKDA; chronic kidney disease, losartan-treated, CKDL. Values are means ± SEM of *n* = 6. * *p* < 0.05 versus C; ^+^ *p* < 0.05 versus CKD; ^#^ *p* < 0.05 versus CKDA.

It has been reported that the renin-angiotensin system (RAS) is involved in CKD progression; thus, we assessed the protein expression of AT1R and AT2R in the renal cortex using immunoblotting assays. In the CKD group, the protein expression of both AT1R and AT2R was reduced in relation to the control group (Figure 5). The allicin treatment prevented the decrease in AT1 expression observed in the untreated CKD group (Figure 5a). However, allicin did not completely restore the levels of AT1R expression. By contrast, there were no changes in the AT1R expression levels between losartan-treated or untreated CKD groups, and even these were lower compared with the allicin treatment group (Figure 5a). On the other hand, allicin treatment restored the AT2 expression levels in contrast to those observed in the untreated CKD group (Figure 5b). Additionally, losartan treatment was able to prevent the downregulation of angiotensin II receptors in the renal cortex from the CKD group (Figure 5). In this context, we did not find differences between the allicin and losartan treatments.

### 2.6. Evaluation of Renal Oxidative Stress

In CKD, there is an increase in pro-oxidant molecules and/or a decrease of antioxidant enzymes. Hence, we studied the oxidative stress using lipid and protein oxidation as markers of oxidative stress and genes under the control of the antioxidant Nrf2/Keap1 system.

In the cortex and medulla from rats with CKD there was an oxidative stress status, as demonstrated by the content of oxidized proteins and lipid peroxidation, which was higher in the CKD group than in the control animals. These results were observed in the cortex as well as in the medulla (Table 2). Allicin treatment was as effective as losartan treatment in reducing the lipid oxidation in the cortex and medulla (Table 2). Concerning the effects of allicin and losartan on oxidized proteins, both treatments reduced the oxidation of proteins in the cortex (Table 2). This effect was not observed in the renal medulla, where only allicin showed antioxidant effects by decreasing protein oxidation.

**Figure 6:**
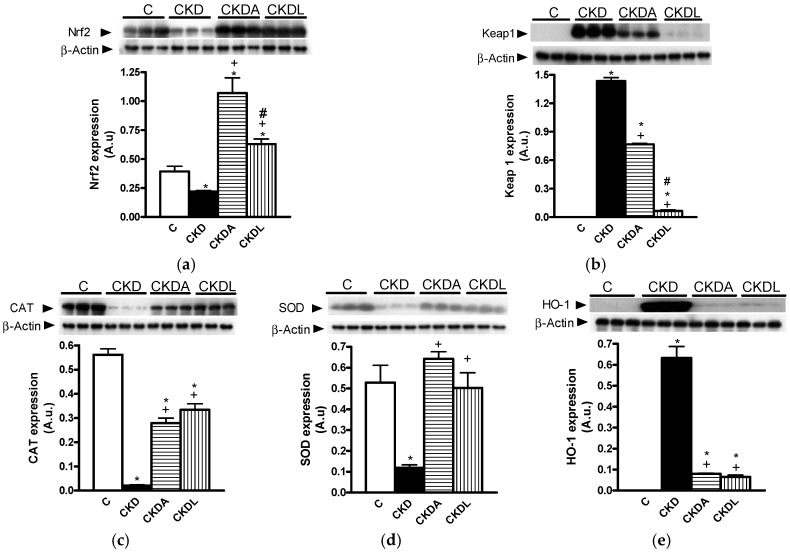
Analysis of antioxidant pathway Nrf2/Keap1 in the renal cortex. (**a**) Nuclear factor (erithroid-derived 2)-like 2 (Nrf2); (**b**) Kelch-like ECH-associated protein 1 (Keap1); (**c**) catalase (CAT); (**d**) superoxide dismutase (SOD); (**e**) heme oxygenase-1 (HO-1). Representative immunoblot and quantitative analysis of relative protein expression. Control, C; chronic kidney disease, CKD; chronic kidney disease, allicin-treated, CKDA; chronic kidney disease, losartan-treated, CKDL. Values are presented as the mean ± SEM of *n* = 6. * *p* < 0.05 versus C; ^+^ *p* < 0.05 versus CKD; ^#^ *p* < 0.05 versus CKDA.

Additionally, we analyzed the expression of Nrf2, Keap1, catalase (CAT), superoxide dismutase (SOD), and HO-1 proteins by immunoblotting. In the cortex from the group with CKD, the expression of Nrf2 was decreased compared with that of the control group. By contrast, the expression of Keap1 was higher in the CKD rats with respect to the control group (Figure 6). The allicin or losartan treatments increased the expression of Nrf2, although both treatments down-regulated Keap1 expression. Nevertheless losartan showed better effectiveness than allicin in the down-regulation of Keap1 (Figure 6). Moreover, the expression of CAT and SOD decreased in the cortexes of the CKD animals. The expression of HO-1 was induced in the CKD group compared with the control group, where the expression of this protein was not observed (Figure 6).

The allicin or losartan treatments restored the down-regulation of CAT and SOD, but the effectiveness of losartan was better than that of allicin (Figure 6). In the case of SOD expression, allicin and losartan were equally effective in restoring the levels of SOD in the cortex (Figure 6). The expression of HO-1 was down-regulated by the allicin or losartan treatments. However, these effects did not reach the levels observed in the control group. We did not observe differences between allicin and losartan in SOD and HO-1 expression levels (Figure 6).

**Figure 7:**
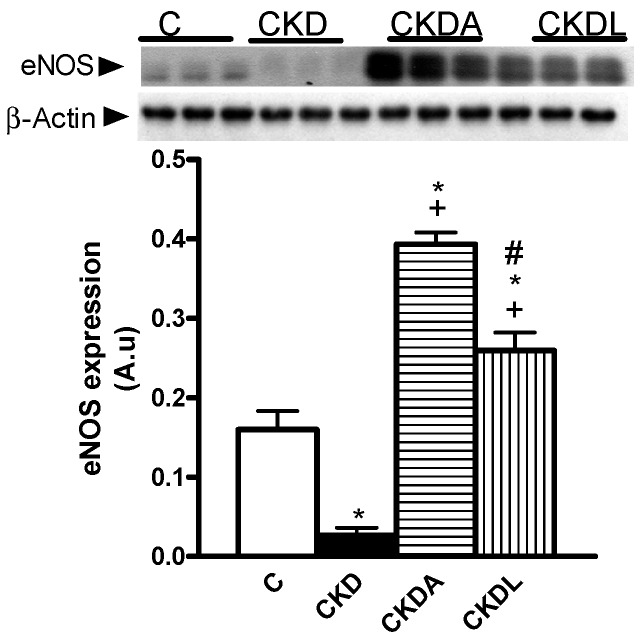
Renal expression of endothelial nitric oxide synthase (eNOS) in the cortex. Representative immunoblot and quantitative analysis of relative protein expression. Control, C; chronic kidney disease, CKD; chronic kidney disease, allicin-treated, CKDA; chronic kidney disease, losartan-treated, CKDL. Values are means SEM of *n* = 6. * *p* < 0.05 versus C; ^+^ *p* < 0.05 versus CKD; ^#^ *p* < 0.05 versus CKDA.

It has been described that hypertension and oxidative stress in the kidneys is associated with an alteration in endothelial nitric oxide synthase (eNOS) expression. To address this issue we assessed the eNOS expression by immunoblotting. In the CKD group, the expression of eNOS was decreased in comparison with the control group (Figure 6). However treatment with allicin or losartan increased the eNOS expression, with allicin being more efficient than losartan (Figure 7).

### 2.7. In Silico Analysis of AT1-Losartan and -Allicin Interactions

**Figure 8:**
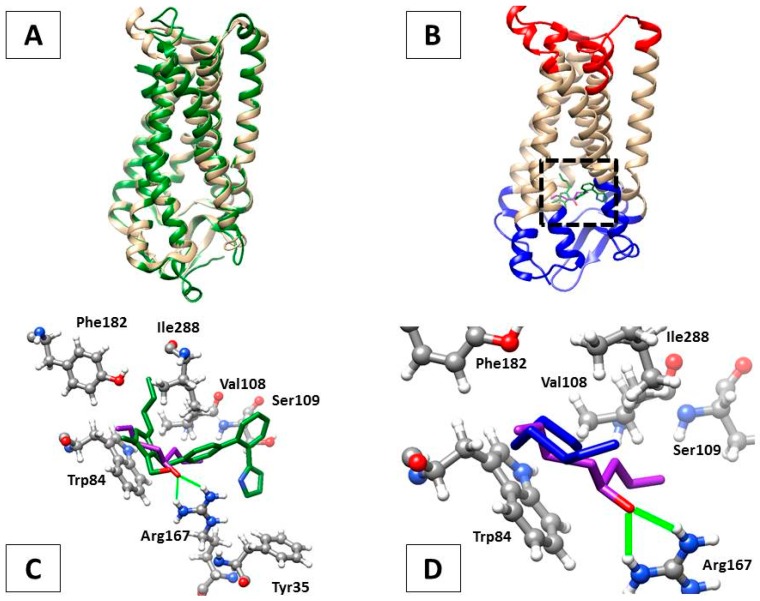
In silico analyses of the interactions of AT1R homologs with losartan and allicin. (**A**) Protein structure alignment of rat (RnAgtR1A, light brown) and human (HsAgtR1, green) DNA, showing their high similarity; (**B**) Docking of allicin (purple) and losartan (green), both in stick representation, at the transmembrane region (dotted square) just beneath the extracellular domain of RnAgtR1A (blue). The intracellular domain of the receptor is shown in red; (**C**) Docking of allicin (purple) and losartan (green), each with the oxygen atom in red, with the putative inhibitor-interacting aminoacids of RnAgtR1A. Note the hydrogen bonds (green lines) formed between oxygen from allicin and H12 and H22 of Arg167; (**D**) Docking of allicin (purple) and its oxygen-free precursor diallyl disulfide (blue) at the niche of putative inhibitor-interacting amino acids, where the bridge-forming oxygen atom of allicin drives a distinct conformation and accommodation of this ligand.

On the basis of the similar degree of overall nephroprotection provided by losartan and allicin treatments at the physiological, histological, functional, and molecular levels in the rat model of CKD, it was conceivable that both compounds could act by a similar mechanism. Since losartan is a first-choice antihypertensive drug that acts by inhibiting the activation of AT1 receptors that, upon stimulation with angiotensinogen II, promote vasoconstriction, we carried out an in silico analysis to assess if allicin could be interacting with AT1 in the same manner. For this, protein-ligand docking predictions and the most favored complex energies (∆G) were obtained using *Rattus norvegicus* angiotensin II receptor type 1A (RnAgtR1A) and *Homo sapiens* angiotensin II receptor type 1 (HsAgtR1) as AT1 receptor homologs and losartan or allicin as ligands. As expected, the structural comparison of human and rat AT1 counterparts showed a striking similarity of 95.6% (the threading metrics (TM) score was 0.9560, normalized with RnAgtR1A: Figure 8A). Interestingly, the most favored docking positions for losartan (∆G = −10.172 kCal/mol) and allicin (∆G = −6.839 kCal/mol) in RnAgtR1A were located at the same transmembrane region, near to the extracellular domain of the receptor, where olmesartan experimentally complexes in the reference crystal structure of human AT1 (Figure 8B, dotted square). As a reference, olmesartan displays its most favored docking with RnAgtR1A at ∆G = −10.158 kCal/mol.

At the sequence level, RnAgtR1A and HsAgtR1 share the seven inhibitor-interacting residues (Tyr35, Trp84, Val108, Ser109, Arg167, Phe182, and Ile288) of which Arg167 is an interesting feature. As can be seen in Figure 8C, the oxygen atom of allicin is able to form hydrogen bonds with H12 and H22 of Arg167 that might contribute to a significant strengthening of the receptor-inhibitor interaction.

**Figure 9:**
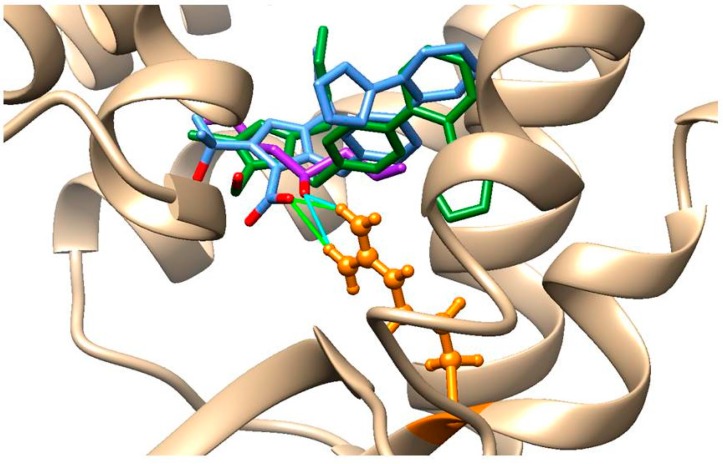
Optimal docking of allicin (purple), losartan (green), and olmesartan (blue), all in stick representation, in RnAgtR1A in which hydrogen bonds are formed by the oxygen atoms O1 of olmesartan (green lines) and of allicin (cyano lines) with hydrogens H12 and H22 from arginine 167 (orange), whilst the unique oxygen atom of losartan is docked more distantly.

Remarkably, the unique oxygen atom of losartan trends directly towards H12 and H22 of Arg167. This notion is further supported by two additional observations: (a) the optimal docking of the oxygen-free precursor of allicin, namely diallyl disulfide (∆G = −6.316 kCal/mol), exhibits a strikingly different conformation that is farthest from Arg167 (Figure 8D) and (b) olmesartan, a hydrolyzable version of a sartan-type AT1 inhibitor, has three oxygen atoms with which O1 is able to form hydrogen bonds, as allicin’s oxygen does (Figure 9).

Altogether these bioinformatics analyses suggest that the comparable nephroprotective effects of losartan and allicin indicate a common mechanism involving AT1 receptors.

## 3. Discussion

Hypertension is a hemodynamic characteristic of CKD and is considered one of the most important factors in determining the rate of deterioration of renal function by worsening glomerular injury and proteinuria [7]. It is believed that hypertension and CKD may be mutually both the cause and consequence of each other [1]. Recently, we reported that allicin treatment improved cardiac dysfunction and decreased hypertension, vascular reactivity to Ang II, and the oxidative stress in a way dependent on the Nrf2/Keap1 system [18]. This previous study showed the therapeutic potential of allicin for a physiopathological condition in which the cardiac and/or renal functions are involved and compromised. However, we did not delve into the study of the effects of allicin on the kidney. Thus, the aim of the present study was to contribute to the knowledge of this issue and, on the other hand, to compare the therapeutic efficacy of allicin with losartan, a well-known and common drug used in the clinic to treat hypertension.

High BP is a hemodynamic characteristic of CKD, which could accelerate the progression of renal dysfunction by worsening glomerular injury and proteinuria which, in turn, promotes further glomerular and tubulointerstitial injury. As a consequence, a fall in the glomerular filtration rate may ensue [7]. Furthermore, albuminuria and urinary excretion of NAG are increased in hypertensive patients and experimental models, and both are frequently used as an index of renal damage, specifically as glomerular and tubular markers, respectively [19,20]. In line with these reports, we observed an increase in systolic blood pressure at the third week of follow-up, which was aggravated at the sixth week after renal ablation, and this was associated with a worsening of glomerular and tubular injury (an increase in albuminuria and urinary NAG).

The main mechanism through which albuminuria occurs in CKD involves the alteration of the structure and function of the glomerular filtration barrier. Nephrin is an important transmembrane protein, fundamental for the podocyte slit diaphragm function due to its regulation of renal filtration by selectively allowing small molecules like ions to pass through, while excluding the passage of large molecules like proteins [21,22]. Our results showed an increase in nephrin expression in the CKD group. Other studies in hypertensive patients and experimental models have described increased nephrin expression in early stages [23,24,25,26]. In fact, in patients with CKD, a loss of nephrin occurs despite the increased level of nephrin mRNA [23]. In contrast, the longer-term studies report that, in the advanced stages of the disease, nephrin expression is reduced [23,24,25,26].

It has been suggested that the up-regulation of nephrin may be compensatory to protect against podocyte injury. However, in the long-term, the mechanism may not be able to counteract the injury, and the downregulation may be a result of podocyte injury, which progressively aggravates renal function and proteinuria.

We hypothesized that hemodynamic forces induced the loss of nephrin in urine, allowing the passage of albumina and other small molecules. To compensate this loss and to maintain the integrity of the glomerular filtration barrier, the kidneys increased the nephrin expression. Our results suggested structural alterations; therefore, we studied the renal histology. The kidney injury was clearly shown by the histologic analysis at the glomerular and tubular levels, which was attenuated by the allicin or losartan treatments at both the biochemical and structural levels.

Allicin and losartan showed the same effectiveness to decrease hypertension, albuminuria, and urinary NAG excretion. The level of blood pressure reduction achieved with allicin was comparable to that of common antihypertensive medication (−15 and −30.2 mmHg over three and six weeks, respectively) with respect to untreated CKD, which showed ≥180 mmHg at the end of the study. The results indicated that allicin had a significant effect in reducing blood pressure, which is consistent with previous studies, even in hypertensive patients [15,16,17].

In addition, the expression of nephrin was decreased with allicin and losartan. We think that, due to the reduction in hemodynamic pressure on the glomerular filtration barrier, this remained stable and the synthesis of nephrin decreased, finally contributing to the improvement of renal function.

The RAS plays a pivotal role in many of the pathophysiological changes that lead to the progression of renal disease. These changes are mediated by the interaction of AngII with its receptors. In this study, we find decreased AT1 and AT2 receptor expression in the kidneys. Other studies have reported a decrease in AT1R and AT2R expression in renal hypertension [20,27,28,29]. It has been hypothesized that the AT1R expression decreased as a protective mechanism to counteract the deleterious effects of hyperactive intrarenal RAS. Moreover, it is well documented that AT1R in blood vessels and the kidneys are under negative feedback modulation by Ang II. High systemic and renal Ang II levels downregulate, whereas the low concentration of plasma and renal Ang II upregulates AT1R in the kidneys [29,30,31]. This downregulation of AT1R expression observed with Ang II stimulation is particularly important in disease states characterized by an activated RAS [29,30]. Allicin and losartan treatment prevents the down-regulation of the CKD induced angiotensin II receptors. To our knowledge, this is the first study in which the effect of allicin on angiotensin receptors in the kidneys is described. Despite the CKD group having reduced AT1 receptor expression, this model is still very sensitive to the renoprotective effects of AT1 receptor antagonists. Moreover, strong support for the likely interaction of allicin with AT1R was provided by in silico modelling and docking analyses in which this garlic derivative optimally complexes at the same site where typical AT1R inhibitors (losartan, olmesartan) promote hypertension blocking and where an oxygen atom from these compounds could form hydrogen bonds (especially with Arg167), thereby strengthening the receptor-inhibitor interactions and pharmacological effects. This last notion would be important in designing drugs to interact with AT1R, as exemplified by olmesartan, an ester prodrug that, upon hydrolysis, displays several oxygen atoms able to form hydrogen bonds with Arg167 in AT1R. However another potential mechanism for allicin nephroprotection might include its free radical-scavenging properties [32], even in CKD, as described below, as well as an inhibitory effect on angiotensinogen converting enzyme (ACE) activity, which has already been reported for garlic extracts in normal and diabetic rats [33].

On the other hand, oxidative stress in CKD and hypertension commonly accompanies both disorders. Our results showed increased lipid and protein oxidation, which are indicators of oxidative stress. We evaluate the expression of antioxidant enzymes as an indicator of balance between the generation and the elimination of free radicals and the expression of Nrf2-Keap1 a master regulator of antioxidant genes. CAT and SOD were decreased. These results were associated with decreased Nrf2, and its repressor, Keap1, increased. In contrast, HO-1 was increased. This can be explained because HO-1 is a protein upregulated by several stimuli such as oxidative stress, nitric oxide, growth factors, cytokines, modified lipids, heat shock, ischemia-reperfusion, and its substrate, heme [34,35]. In addition, the HO-1 promoter contains binding sites for nuclear factor-kappa B (NFκB), transcription factor activator protein-1 (AP-1), cAMP response element binding protein (CREB), antioxidant response element (ARE), and Nrf2 binding. On the other hand, several signaling pathways that include protein kinase C (PKC) or protein kinase A (PKA) and extracellular signal regulated kinase (ERK)/mitogen-activated protein kinase (MAPK) may also regulate HO-1 at the translational level rather than at the transcriptional level [34,35].

Both the allicin and the losartan treatments were effective in reducing alterations in CKD-induced oxidative status in a dependent pathway of Nrf2/Keap1.

It has been reported that allicin can induce the Nrf2/Keap1 system [18,36], which could stimulate the expression of antioxidant enzymes and decrease oxidative stress. However, to our knowledge, this is the first study that reports the effect of allicin on the Nrf2/Keap1 pathway in the kidneys, especially in the context of an emerging disease such as CKD.

Additionally, we assessed the eNOS expression as an indicator of endothelial function. Our result showed decreased eNOS in the kidney, thus suggesting an alteration in vasodilation, which could contribute to increased blood pressure. These findings are in agreement with those reported in other studies [37,38,39]. The allicin or the losartan treatment increased the eNOS expression, but allicin was more effective than losartan. In CKD, the excess of ROS may directly stimulate vascular contraction or reduce nitric oxide, contributing to hypertension [40]. Moreover, there is an increase of asymmetric dimethylarginine (ADMA), an inhibitor of nitric oxide synthase [41]. These two factors are responsible for a decrease in nitric oxide activity in CKD, which contribute to endothelial dysfunction, vasoconstriction, and, therefore, increased blood pressure.

Several reports indicate that allicin byself shows antioxidant properties by scavenging free radicals [42] and hydroxyl radicals (OH•) [43]. Interestingly, Liu et al. [44] described that allicin reduces AngII-induced oxidative stress in cardiac hypertrophy in both in vitro and in vivo models. Other studies have demonstrated that allicin prevents ROS damage by upregulating the phase II detoxifying enzymes and increasing the cellular glutathione levels [45].

Given the well-documented role of ROS in hypertension, it is reasonable to propose that allicin ameliorates hypertension by inhibiting cellular oxidative stress.

On the other hand, it has been suggested that allicin acts as an antihypertensive mainly due to decomposing rapidly to its degradation products. On the other hand, the vasoactivity of garlic compounds is synchronous with hydrogen sulfide (H_2_S) production, and their potency to mediate relaxation increases with (H_2_S) yield, a potent gaseous signaling molecule with the capacity to control blood pressure by the relaxation of smooth muscle cells [46]. In anesthetized casts with intact chests and the isolated lungs of rat under constant flow conditions, allicin showed vasodilatory activity at the systemic and pulmonary levels [47]. Allicin rapidly decomposes, mainly to diallyl sulfide (DAS), diallyl disulfide (DADS), diallyl trisulfide (DATS), and ajoene [13]. However, after consumption, neither allicin nor its metabolites have been found in blood or urine [48], suggesting that the metabolites of allicin are rapidly metabolized.

Altogether, the evidence from the present study suggests that the nephroprotective effects of allicin may be partly due to antihypertensive and antioxidant effects, which preserve the function and renal structures in hypertensive rats with CKD induced by 5/6 Nx.

## 4. Methods

### 4.1. Reagents

Diphenyl dichloromethane, diallyl disulphide, hydrogen peroxide, 4-nitrophenol, methanesulfonic acid, tetramethoxypropane, dinitrophenylhydrazine, 1-methyl-2-phenylindole 4-nitrophenyl-*N*-acetyl-β-d-glucosaminide, acetonitrile, methanol, and guanidine were purchased from Sigma (St. Louis, MO, USA). The anti-angiotensin II type 1 receptor antibody (ab124734) and anti-angiotensin II type 2 receptor antibody (ab92445) were purchased from Abcam (Cambridge, MA, USA). The β-actin antibody GTX109639, eNOS antibody GTX54637, KEAP1 antibody GTX60660, and NRF2 antibody GTX103322 were purchased from GeneTex Inc. (Irvine, CA, USA). The nephrin antibody ALX-810-016-R100 was purchased from Enzo Life Sciences Inc. (Farmingdale, NY, USA). The catalase antibody sc-34281, heme oxygenase 1 antibody sc-1797, and superoxide dismutase antibodysc-8637 were purchased from Santa Cruz Biotechnology Inc. (Dallas, TX, USA). All other chemicals used herein were of the highest analytical grade available.

### 4.2. Experimental Design

Male Wistar rats weighing 280 to 300 g were used. The rats were divided into four groups (*n* = 12 each): sham-operated animals served as a control (C), chronic kidney disease (CKD) (induced by renal ablation (5/6 nephrectomy)), CKD+allicin (40 mg/kg/day pathway oral) (CKDA), and CKD+Losartan (20 mg/kg/day pathway oral) (CKDL). All experimental groups were fed with commercial rodent pellets (PMI Nutrition International, Inc., LabDiet 5008, Richmond, IN, USA) and water ad libitum for six weeks.

### 4.3. Ethics Statement

This study was performed in accordance with the Guide for the Care and Use of Laboratory Animals, published by the U.S. National Institute of Health and approved by the Research Committee of the National Institute of Cardiology Ignacio Chávez and by the Mexican Federal Regulation for animal experimentation and care (NOM-062-ZOO-2001) and for the disposal of biological residues (NOM-087-ECOL-1995).

### 4.4. Allicin

Allicin (diallyl thiosulfinate) was produced by the oxidation of diallyl disulphide, as previously reported [32]. In brief, 1 g diallyl disulphide was dissolved in 5 mL acetic acid under stirring in an ice bath. Hydrogen peroxide (1.5 mL, 30% *v*/*v*) was added stepwise, and the reaction was allowed to proceed for 30 min. Afterwards, the reaction was kept at 13 °C for 20 min; then it was put in an ice bath again for 5 h, stopped with 15 mL distilled water at pH 6.5, and extracted with 30 mL dichloromethane. After five extractions with 5% (*w*/*v*) Na_2_CO_3_ (20 mL each) and three extractions with distilled water (20 mL each), the solvent was left to evaporate until a yellowish oil (allicin) remained. For stabilization and storage, allicin was resuspended in water at 2.5% (*w*/*v*) and kept at –70 °C until used.

### 4.5. Induction of Experimental Model of Chronic Kidney Disease

Under deep anesthesia with isofluorane, subtotal renal ablation was performed by the removal of the right kidney and the selective infarction of approximately two thirds of the left kidney by ligation of two or three branches of the renal artery (5/6 Nx). The sham operation consisted of ventral laparotomy and the manipulation of the kidneys and renal pedicle without the destruction of renal tissue. The muscle and skin incisions were sutured with polypropylene suture. After surgery, the animals were returned to the animal facility for recovery [18].

### 4.6. Systolic Blood Pressure (SBP)

SBP was measured in conscious restrained rats by tail-cuff plethysmography (XBP-1000 Kent Scientific, Torrington, CT, USA). The rats were preconditioned twice before SBP was measured at the basal period; the mean of five consecutive readings was recorded as the blood pressure, three and six weeks after renal ablation.

### 4.7. Renal Function

Three and six weeks after renal ablation, the rats were placed in metabolic cages (Nalgene, Rochester, NY, USA) to collect 24-h urine. Urine samples were centrifuged at 5000× *g* for 15 min to remove debris, and the supernatant was analyzed. The variables measured were creatinine and blood urea nitrogen (BUN) (IL 300 plus, Clinical Chemistry Analyzer, Instrumentation Laboratory, Bedford, MA, USA), microalbuminuria (Albumin Rat ELISA Kit, Abcam Cambridge, MA, USA), and urinary *N*-acetyl-β-d-glucosaminidase (NAG) activity (spectrophotometric assay).

### 4.8. Measurement of NAG Activity

For the determination of NAG activity in urine samples, 4-nitrophenyl-*N*-acetyl-β-d-glucosaminide was used as the substrate. One unit of enzymatic activity (U) represents the amount of enzyme that hydrolyses one mol of substrate per min at 37 °C. The results were expressed as units/day (U/24 h).

To evaluate the tubular injury, a set of animals was sacrificed after three weeks of follow-up after renal ablation (six rats per group). The remaining rats were studied after six weeks of follow-up (six rats per group). All experimental groups were maintained with a laboratory diet and water ad libitum.

The rats were euthanized by anesthesia with isoflurane and exsanguination. A blood sample was collected and centrifuged. The plasma was frozen until used in further analyses. The kidney was excised, washed with cold phosphate-buffered saline, and longitudinally divided into two sections: one part for protein work, which was excised and divided into cortex and medulla, frozen in liquid nitrogen, and stored until further processing, and the other for hematoxylin and eosin (H and E) staining, which was fixed in 10% formalin in phosphate-buffered saline (PBS) and embedded in paraffin for histological studies.

### 4.9. Histopathological Study

From the paraffin blocks, 4 µm sections were obtained, hematoxylin and eosin stained (HE), and observed with a Zeiss Axiophot2 light microscope. All slides were analyzed in a blinded fashion. For a fibrosis assessment, 10 cortex field images (200× magnification) were recorded at random from Sirius Red-stained sections of each kidney. The evaluation of fibrosis focused on red-stained areas (collagen I and collagen III) located between tubules and glomeruli, and its quantification was performed with a computerized image analysis software (Axiovision Rel. 4.8.2, Zeiss, Jena, Germany). Microscopic images were obtained using a digital camera mounted on a light microscope (Axiophot 2, Zeiss, Jena, Germany). All slides were analyzed in a blinded fashion.

### 4.10. Evaluation of Renal Oxidative Stress

#### 4.10.1. Determination of Lipid Peroxidation

4-Hydroxynonenal (4-HNE) was measured using a standard curve of tetramethoxypropane. A solution of 1-methyl-2-phenylindole in acetonitrile:methanol (3:1) was added to heart homogenates, and the reaction was started with 37% HCl or methanesulfonic acid plus FeCl_3_ to measure 4-HNE. The optical density was measured at 586 nm after 1 h of incubation at 45 °C. The data were expressed as nmol of 4-HNE per milligram of protein (nmol HNE/mg protein).

#### 4.10.2. Measurement of Oxidized Proteins

The presence of carbonyl groups in the proteins was measured using the reaction with 2,4-dinitrophenylhydrazine (DNPH). The protein carbonyl groups were estimated by using the molar absorption coefficient of 22,000 M^−1^∙cm^−1^ for DNPH derivatives, and their concentrations were expressed as nmol carbonyl groups/mg protein. Guanidine solution was used as a blank.

#### 4.10.3. Expression of Nephrin, Nrf2/Keap1 Pathway, Endothelial Nitric Oxide Synthase (eNOS), and Angiotensin II Receptors in Renal Cortex

The cortex was washed thoroughly with ice sold saline 10% (*w*/*v*) and later homogenized in a Potter Elvehjem homogenizer in ice-cold 50 mM phosphate buffer at pH 7.4 containing mammalian protease inhibitor cocktail (Halt™ Protease Inhibitor Cocktail, Thermo Fisher Scientific, Waltham, MA, USA). The homogenates were used for the determination of total protein concentration by the Bradford method using bovine serum albumin as the standard. Equal protein concentrations (15 µg) were denatured in gel loading buffer at 85 °C for 5 min and then loaded onto 10% sodium dodecyl sulfate polyacrylamide gel electrophoresis and transferred to polyvinylidene difluoride (PVDF) membranes and incubated at 4 °C overnight with the primary antibody diluted in phosphate buffered saline with the detergent Tween^®^ 20. Housekeeping protein β-actin was used as a loading control. Positive immunoreactive bands were quantified densitometrically and expressed as the ratio of problem to β-actin in arbitrary units. The protein bands were visualized with enhanced chemiluminescence reagents (ECL Plus Western Blotting Detection System, Amersham Pharmacia Biotech, Piscataway, NJ, USA) and analyzed, and their intensity was quantified using a Kodak Electrophoresis Documentation and Analysis System 290 (EDAS 290).

### 4.11. Bioinformatics Analyses

In order to predict the ability of typical angiotensin II receptor inhibitors (losartan, olmesartan) and garlic compounds (allicin and diallyl diulfide) to interact with, and thereby inhibit, the function of rat (RnAgtR1A) and human (HsAgtR1) counterparts of the angiotensin II receptor, in silico approaches of molecular docking were performed using the Swiss Dock [49] web service. This tool first calculates, orders in clusters, and delivers a set of 256 viable positions between one rigid target (protein) and one flexible ligand (compound), which are input in Protein Data Bank (PDB) and Tripos molecule structure (MOL2) formats, respectively. Of these predictions, the most favored docking was selected by considering the lowest (i.e., most negative value) Gibbs free energy (∆G). In these analyses, the protein structures of RnAgtR1A and HsAgtR1 were raised using the Swiss Model server [50], whilst the crystal structure of human angiotensin receptor II type 1 (PDB ID: 4YAY, ligand: olmesartan) was used as the structure of reference to identify inhibitor-interacting amino acids. All predictions obtained from SwissDock were visualized and edited using the University of California at San Francisco (UCSF)-Chimera package v. 1.10.1 (UCSF, San Francisco, CA, USA). Structural comparisons of the modelled proteins were carried out using the TM-Align tool accessible via the I-TASSER server [51].

### 4.12. Statistical Analyses

All the values are expressed as mean ± standard error of the mean (SEM). Statistical significance was determined by one-way ANOVA, followed by Bonferroni analysis using Prism 5 (GraphPad, San Diego, CA, USA) software. Differences were considered significant when *p* < 0.05.

## 5. Conclusions

Allicin showed antihypertensive, antioxidant, and nephroprotective effects. The beneficial effects showed by allicin are similar to or better than those exerted by losartan. In fact, the effect of allicin on blood pressure and renal function is comparable to reductions seen with losartan, a prescription drug commonly used as a first-line therapy. Therefore, allicin may be a useful tool for the treatment of emerging diseases such as CKD.

## Figures and Tables

**Table 1:** The physiologic characteristics of experimental groups.

	Control	CKD	CKDA	CKDL
Body weight (g)	376.53 ± 2.38	331.69 ± 30.81 *	364.45 ± 16.94 ^+^	370.6 ± 6.05 ^+^
Serum creatinine (mg/dL)	0.42 ± 0.02	1.43 ± 0.17 *	1.122 ± 0.10 *^,+^	0.825 ± 0.28 *^,+,#^
Creatinine clearance (mL/min)	1.42 ± 0.12	0.751 ± 0.09 *	1.05 ± 0.10 *^,+^	1.19 ± 0.09 *^,+,#^
Blood urea nitrogen (mg/dL)	4.16 ± 0.40	63.16 ± 10.96 *	42.18 ± 6.21 *^,+^	38.66 ± 3.77 *^,+^

CKD: chronic kidney disease; CKDA: chronic kidney disease, allicin-treated; CKDL: chronic kidney disease, losartan-treated. Data represent the mean ± SEM, *n* = 6. * *p* < 0.05 versus control; ^+^ *p* < 0.05 versus CKD; ^#^ *p* < 0.05 versus CKDA.

**Table 2:** Markers of oxidative stress.

	C	CKD	CKDA	CKDL
Oxidized proteins in the cortex (DNPH nmol/mg protein)	0.90 ± 0.30	122.2 ± 20.99 *	59.87 ± 4.06 *^,+^	76.7 ± 6.29 *^,+^
Oxidized proteins in the medulla (DNPH nmol/mg protein)	1.09 ± 0.30	74.42 ± 1.82 *	36.27 ± 5.19 *^,+^	63.04 ± 3.46 *^,+^
Lipid peroxidation in the cortex (4HNE nmol/mg protein)	1.27 ± 0.28	6.18 ± 0.32 *	2.26 ± 0.4 ^+^	2.04 ± 0.88 ^+^
Lipid peroxidation in the medulla (4HNE nmol/mg protein)	1.06 ± 0.36	6.13 ± 1.12 *	1.88 ± 0.28 ^+^	0.92 ± 0.21 ^+^

C: control; CKD: chronic kidney disease; CKDA: chronic kidney disease, allicin-treated; CKDL: chronic kidney disease, losartan-treated. Data represent the mean ± SEM, *n* = 6. * *p* < 0.05 versus control; ^+^ *p* < 0.05 versus CKD; ^#^ *p* < 0.05 versus CKDA.
